# 1-Acetyl-β-Carboline from a Jeju Gotjawal Strain *Lentzea* sp. JNUCC 0626 and Its Melanogenic Stimulating Activity in B16F10 Melanoma Cells

**DOI:** 10.3390/molecules29194586

**Published:** 2024-09-27

**Authors:** Kyung-A Hyun, Yang Xu, Kyung-Hwan Boo, Chang-Gu Hyun

**Affiliations:** 1 Department of Biotechnology, College of Applied Life Science, Jeju National University, Jeju 63243, Republic of Korea; kah990626@gmail.com; 2 Department of Beauty and Cosmetology, Jeju Inside Agency and Cosmetic Science Center, Jeju National University, Jeju 63243, Republic of Korea; iamxuyang1990@gmail.com

**Keywords:** 1-acetyl-β-carboline, *Lentzea* sp., melanogenesis, B16F10 melanoma cells

## Abstract

The genus *Lentzea* is a prolific source of bioactive and structurally diverse secondary metabolites. We isolated a novel strain, *Lentzea* sp. JNUCC 0626, from Hwasun Gotjawal on Jeju Island, Korea. Based on 16S rRNA partial gene sequence analysis, strain JNUCC 0626 is closely related to *Lentzea isolaginshaensis* NX62 (99.41% similarity), *Lentzea pudingi* DHS C021 (99.31%), and *Lentzea cavernae* SYSU K10001 (99.26%). From the fermentation broth of JNUCC 0626, we isolated 1-acetyl-β-carboline, whose structure was established using IR, HR-ESI-MS, and 1D- and 2D-NMR techniques. 1-acetyl-β-carboline was found to activate melanogenesis in mouse B16F10 cells without cytotoxicity at concentrations up to 50 μM. At this concentration, the compound increased melanin content by 27.44% and tyrosinase activity by 240.64% compared to the control, by upregulating key melanogenic enzymes, including tyrosinase, TRP-1, TRP-2, and microphthalmia-associated transcription factor (MITF), a central regulator of melanogenesis. In addition, 1-acetyl-β-carboline significantly inhibited ERK phosphorylation, reducing it by 20.79% at a concentration of 12.5 μM and by 25.63% at 25 μM. This inhibition supports the hypothesis that 1-acetyl-β-carboline enhances melanin synthesis by upregulating MITF and melanogenic enzymes via the ERK signaling pathway. This study aimed to isolate and identify 1-acetyl-β-carboline from a novel strain of *Lentzea* sp. JNUCC 0626, discovered in Gotjawal, Jeju Island, and to evaluate its effect on melanin production in B16F10 melanoma cells. Skin irritation tests on 32 subjects confirmed its safety for topical use, and the findings suggest that 1-acetyl-β-carboline, which enhances melanogenesis without cytotoxicity, holds promise as a therapeutic agent for hypopigmentation-related conditions or as a cosmetic ingredient.

## 1. Introduction

Exploring the diversity of natural chemicals is a crucial strategy for drug discovery, as natural products often exhibit highly potent and selective biological activities. During the golden age of antibiotic discovery, *Streptomyces* demonstrated an unparalleled capacity to produce bioactive metabolites [1,2]. However, this success was hindered by the issue of rediscovery. To overcome this issue, researchers can explore rare actinomycetes—bacteria that are part of the order Actinomycetales but do not belong to the genus Streptomyces—as a potential source for discovering novel biologically active compounds [3]. Although these genera are rarely isolated through standard laboratory methods, increasing evidence suggests that they possess significant biosynthetic and chemical diversity [4,5]. The term “rare” actinomycetes refers to the artificial classification of all actinomycete genera, excluding the genus *Streptomyces*. Taxa included in this classification are often ambiguous [6,7]. The genus *Lentzea* is regarded as a rare actinomycete. An increasing number of novel biologically active metabolites, such as antibiotics and cytotoxic compounds, have been discovered within this genus [8,9].

Actinomycetes also have great potential as cosmetic ingredients. Among them, the *S. chattanoogensis* THA-663S strain is particularly notable for its anti-aging and antibacterial properties. The ethyl acetate extract of this strain protects UVB-irradiated HaCaT keratinocytes and inhibits the proliferation of methicillin-resistant *Staphylococcus aureus* (MRSA). Consequently, THA-663S effectively mitigates skin cell aging and counters aging induced by pathogenic infections [10]. Furthermore, actinomycetes have proven efficacy in the treatment of dermatological conditions such as melanin dysregulation [11,12,13], psoriasis [14], atopic dermatitis [15], and acne [16,17]. Collectively, these findings underscore the potential of actinomycete-derived compounds as valuable cosmetic ingredients with diverse skin care benefits.

The term “Gotjawal” derives from two Korean words: “Got”, which means forest, and “Jawal”, which means dense, bushy vegetation. Gotjawal refers to a unique forest ecosystem on Jeju Island, characterized by thick, lush vegetation that grows in volcanic lava fields [18]. Gotjawal is formed on volcanic lava fields and supports a high level of biodiversity, including various actinomycetes [19,20,21]. These bacteria thrive in porous, nutrient-rich volcanic soils, contributing to nutrient cycling and organic matter decomposition [22,23]. Ultimately, Gotjawal supports a rich diversity of actinomycetes, whose capacity to produce biologically active compounds makes them prime candidates for industrial research. Thus, exploring and isolating Gotjawal actinomycetes is crucial to discovering new bioactive substances with potential industrial and pharmaceutical applications, highlighting the importance of microbial research in this ecosystem [24,25].

This study isolated a new *Lentzea* strain, designated JNUCC 0626, from the Hwasun Gotjawal soil of Jeju Island, which exhibits remarkable melanogenic activating activity. Furthermore, 1-acetyl-β-carboline was identified as having melanogenic stimulating activity, and its underlying mechanisms in B16F10 melanoma cells were investigated. Finally, the safety of 1-acetyl-β-carboline was assessed through skin irritation tests, which indicated its potential as a protective agent against hypopigmentation for topical applications.

## 2. Results and Discussion

### 2.1. Phylogenetic Analysis

**Figure 1:**
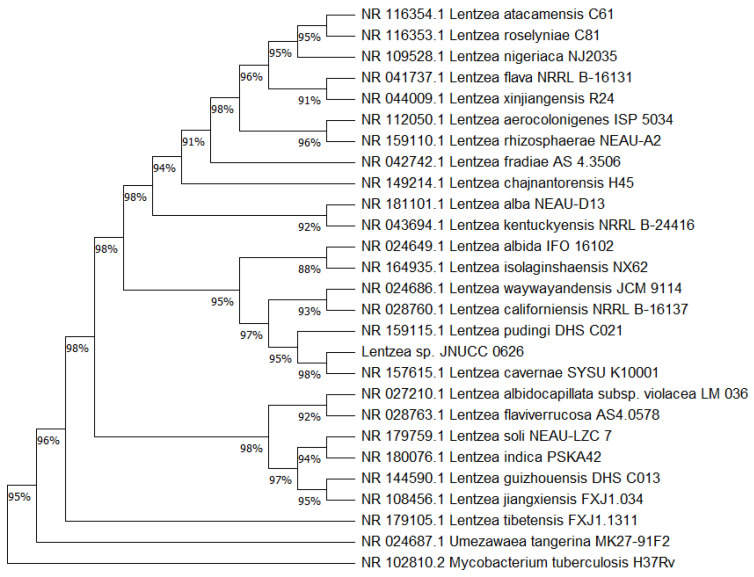
Phylogenetic tree of neighboring based on 16S rRNA partial gene sequence (1480 bp) of *Lentzea* sp. JNUCC 0626. The relationship between closely related species of type cultures from the genus *Lentzea* is demonstrated. *Mycobacterium tuberculosis* H37RvT was used as an outgroup. The percentage of replicate trees in which the associated taxa clustered together in the bootstrap test (1000 replicates) are shown next to the branches in decimal form. Analysis performed using MEGA 11 showed that *Lentzea cavernae* SYSU K10001 is its closest neighbor.

Comparative analysis using the 16S rRNA gene sequence (1480 bp) of strain JNUCC 0626 and the 16S rRNA gene sequences of strains from the EzBioCloud database and GenBank/EMBL/DDBJ database revealed that the isolate was closely related to *Lentzea isolaginshaensis* strain NX62 (NR_164935.1, 99.41%), *Lentzea pudingi* strain DHS C021 (NR_159115.1, 99.31%), *Lentzea cavernae* strain SYSU K10001 (NR_157615.1, 99.26%), and *Lentzea alba* strain NEAU-D13 (NR_181101.1, 98.99%). Phylogenetic trees were constructed using the maximum likelihood (ML) and neighbor-joining (NJ) algorithms, with the analyses performed using MEGA 11 software [26]. Bootstrap values were analyzed with 300 or 1000 replications. Phylogenetic analysis based on 16S rRNA gene sequences showed that strain JNUCC 0626 belonged to the genus *Lentzea* within the Pseudonocardiaceae family (Figure 1).

### 2.2. Isolation and Structural Identification of Compounds

**Figure 2:**
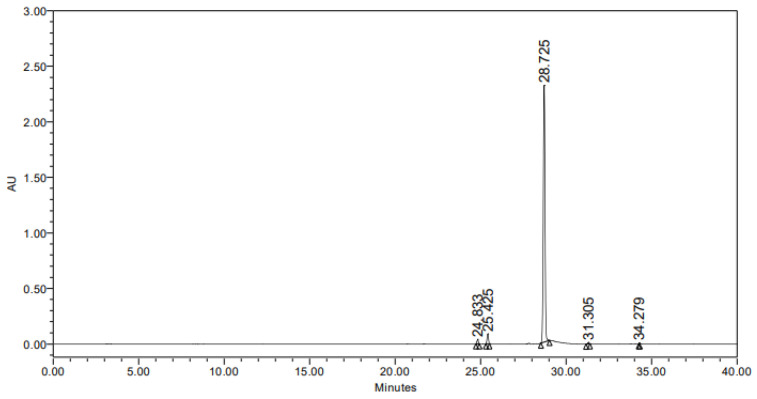
HPLC chromatogram of the isolated compound (UV 283.3 nm).

HPLC analysis was conducted to characterize the compounds present in the hexane extract of *Lentzea* sp. JNUCC 0626. As shown in Figure 2, we identified a single prominent peak with 95.09% purity based on HPLC retention time and UV–vis spectral data. The chromatogram, recorded at 283.3 nm, revealed the peak at a retention time of 28.67 min. Subsequent NMR analysis was performed to elucidate the chemical structure of the isolated compound. By interpreting the NMR data and consulting the relevant literature, the compound was determined to be 1-acetyl-β-carboline: 1H NMR (500 MHz, CDCl_3_) δ 10.42 (s, 1H), 8.62 (d, J = 5.1 Hz, 1H), 8.26 (d, J = 5.1 Hz, 1H), 8.19 (d, J = 7.9 Hz, 1H), 7.65 (dt, J = 15.2, 8.0 Hz, 2H), 7.42–7.34 (m, 1H), 1.25 (s, 3H).13C NMR (126 MHz, CDCl_3_) δ 141.84, 135.30, 130.16, 122.20, 121.29, 120.36, 119.19, 112.20, 26.64.

### 2.3. Stimulatory Effect of JNUCC 0626 Extracts on Melanogenesis in B16F10 Cells

**Figure 3:**
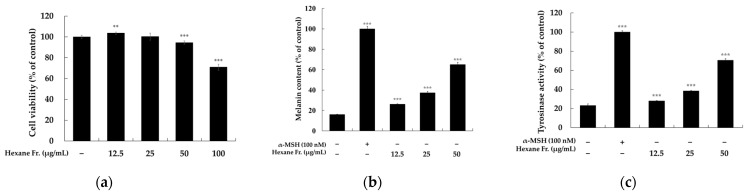
**Effects of hexane extract from *Lentzea* sp. JNUCC 0626 on B16F10 melanoma cells**. (**a**) Cell viability was measured after exposure to the *Lentzea* sp. hexane extract. JNUCC 0626. (**b**) The hexane extract notably promoted melanin synthesis in B16F10 cells. (**c**) Additionally, the extract significantly increased the activity of cellular tyrosinase. Results are expressed as mean ± SD (*n* = 3). Statistical significance was denoted by the following markers: *** *p* < 0.001 and ** *p* < 0.01 compared to the unstimulated control group.

Melanogenesis is a biochemical process initiated by the rate-limiting enzyme tyrosinase, which catalyzes the conversion of tyrosine and facilitates two subsequent key reactions. Tyrosinase-related protein 1 (TRP1) catalyzes the oxidation of 5,6-dihydroxyindole-2-carboxylic acid (DHICA) to its carboxylated indole quinone form. Tyrosinase-related protein 2 (TRP2), also known as dopachrome tautomerase (DCT), converts dopachrome into DHICA. Therefore, activation of tyrosinase is considered a promising strategy for promoting melanogenesis or treating conditions related to hypopigmentation [27,28,29]. To evaluate the potential melanogenic effects of the hexane and ethyl acetate fractions derived from fermentation cultures, we evaluated the melanin content and cellular tyrosinase activity in B16F10 melanoma cells. The impact of JNUCC 0626 hexane fractions on cell viability was assessed using the MTT assay. As shown in Figure 3a, JNUCC 0626 was tested at concentrations ranging from 12.5–100 µg/mL, with cells incubated for 72 h. At concentrations < 100 µg/mL, cell viability remained > 90% compared with untreated controls. Consequently, further investigations were conducted on the effects of JNUCC 0626 on melanin production and tyrosinase enzyme activity at concentrations < 100 µg/mL. JNUCC 0626 significantly increased melanin content and tyrosinase activity in B16F10 cells in a concentration-dependent manner, as illustrated in Figure 3b,c. At a concentration of 50 µg/mL, JNUCC 0626 increased the melanin content and the tyrosinase activity by 303.66% and 204.49%, respectively.

### 2.4. Stimulating Effect of 1-Acetyl-β-Carboline on Melanogenesis in B16F10 Cells

**Figure 4:**
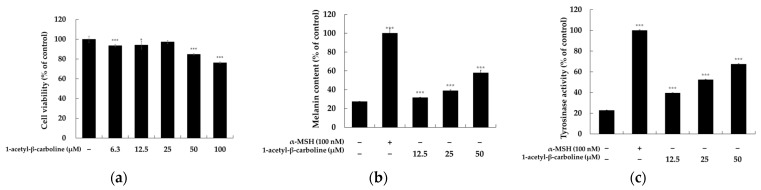
**Effect of 1-acetyl-β-carboline on cell viability, melanin content, and cellular tyrosinase activity in B16F10 cells.** (**a**) Cell viability was assessed after treatment with 1-acetyl-β-carboline. (**b**) 1-acetyl-β-carboline significantly increased melanin synthesis in B16F10 cells. (**c**) Tyrosinase activity was enhanced by 1-acetyl-β-carboline. Data are presented as mean ± SD (*n* = 3). Statistical significance was determined as follows: *** *p* < 0.001 and * *p* < 0.05 compared to the unstimulated control group.

1-acetyl-β-carboline has been identified as a metabolite derived from 7-dehydroxylating enteric bacteria of the bile acid by actinomycetes such as *Streptomyces aculeolatus* MS1–6, endophytic fungi such as *Aspergillus fumigatus* HQD24, and *Lactobacillus* culture broths [30,31,32,33]. This study isolated this compound from *Lentzea* sp. Recent research on 1-acetyl-β-carboline has shown that it inhibits the yeast-to-filament transition of *Candida albicans* by targeting Yak1 [34,35] and exhibits antibacterial activity against MRSA [36]. Despite these findings, there has been no investigation into the skin health benefits of 1-acetyl-β-carboline, particularly in relation to melanogenesis and inflammation. Therefore, we explored its role in melanogenesis to assess the potential application of 1-acetyl-β-carboline in dermatological treatments. The effects of 1-acetyl-β-carboline on melanogenesis in B16F10 cells were thoroughly evaluated. Cell viability was first assessed using the MTT assay after treatment with concentrations ranging from 3.2–50 µM for 72 h. At concentrations < 100 µM, cell viability remained > 90%, indicating low cytotoxicity. Thus, subsequent analyses focused on the effects of 1-acetyl-β-carboline on melanin production and tyrosinase activity. 1-acetyl-β-carboline significantly enhanced both the melanin content and the tyrosinase activity in a concentration-dependent manner (Figure 4b,c). At the highest concentration (50 µM), melanin content increased by 27.44%, while the tyrosinase activity increased by 240.64% compared to the control. Thus, 1-acetyl-β-carboline has potential as a therapeutic agent for the treatment of melanin-related disorders by promoting melanin synthesis and cellular tyrosinase activity.

### 2.5. Effect of 1-Acetyl-β-Carboline on Microphthalmia-Associated Transcription Factor and Melanogenic Enzymes

**Figure 5:**
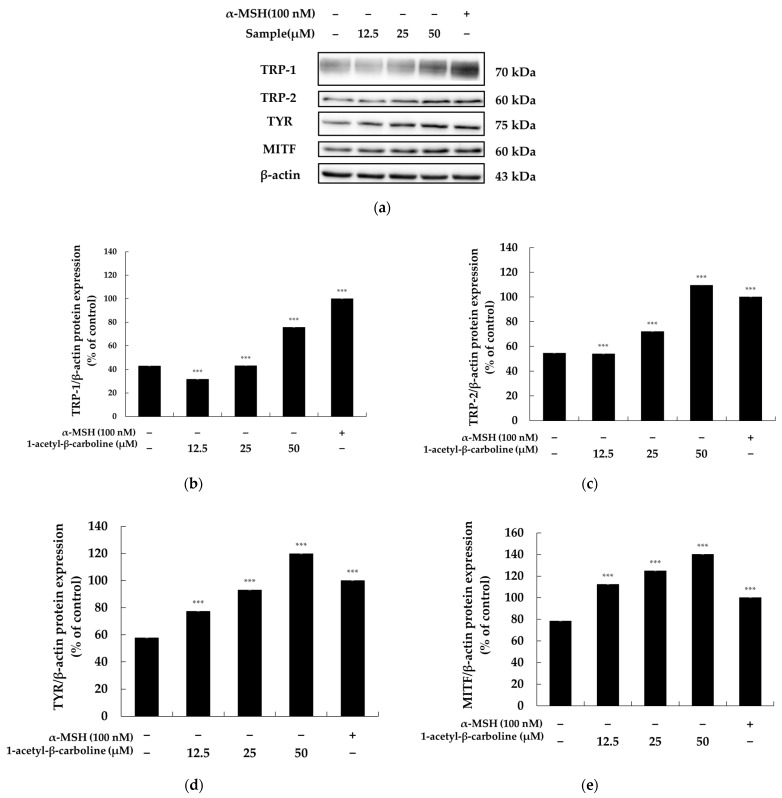
**Effect of 1-acetyl-β-carboline on MITF and melanogenic protein expression in B16F10 cells.** The cells were treated with 1-acetyl-β-carboline (12.5, 25, and 50 μM) for 24 h. (**a**) Western blotting results, the protein expression of (**b**) TRP-1 relative to β-actin, (**c**) TRP-2 relative to β-actin, (**d**) TYR relative to β-actin, and (**e**) MITF relative to β-actin are shown. α-MSH was used as the positive control. Data are presented as mean ± SD (*n* = 3). Statistical significance is indicated as *** *p* < 0.001 vs. unstimulated control.

Microphthalmia-associated transcription factor (MITF), a key regulator of genes involved in cell differentiation, proliferation, and survival, also governs the expression of melanogenic enzymes such as TRP-1 and TRP-2, which are critical for melanin synthesis in melanocytes [37,38]. To explore the pigmentation mechanism of 1-acetyl-β-carboline in melanocytes, its effects on the expression of melanogenic enzymes, including tyrosinase and TRP-2, were examined in B16F10 cells. After treatment with 1-acetyl-β-carboline, the protein levels of tyrosinase and TRP-2 were assessed through Western blot analysis. Compared to untreated controls, 1-acetyl-β-carboline significantly increased the expression of melanogenic enzymes, particularly at a concentration of 50 µM: tyrosinase levels increased by approximately 69.34%. Simultaneously, TRP-1 and TRP-2 levels increased by 84.68% and 113.08%, respectively. Furthermore, MITF expression was markedly up-regulated in a concentration-dependent manner, with activation increasing by approximately 93.56% at higher concentrations (Figure 5). These findings confirm that 1-acetyl-β-carboline enhances TRP-1 levels and promotes the expression of tyrosinase and TRP-2 by modulating MITF expression in B16F10 cells, thus stimulating melanogenesis.

### 2.6. Effect of 1-Acetyl-β-Carboline on the Extracellular Signal-Regulated Kinase (ERK) Signaling Pathway

The MAPK family, including ERK, JNK, and p38 MAPK, plays a crucial role in melanogenesis, though the impact of MAPK/ERK pathway regulation remains debated [39]. Some natural compounds increase melanogenesis by upregulating p-ERK in B16 melanoma cells, while others suggest that elevated p-ERK inhibits melanin synthesis [29,40,41]. This duality may be explained by phosphorylation, which boosts MITF’s transcriptional activity but also triggers its degradation via the ubiquitin-proteasome pathway [42]. 

**Figure 6:**
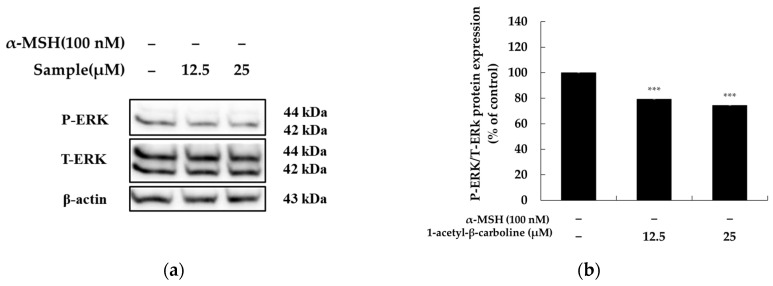
**Effect of 1-acetyl-β-carboline on ERK protein expression in B16F10 cells**. The cells were treated with 1-acetyl-β-carboline (12.5 and 25 μM) for 24 h. Western blot (**a**) and densitometric (**b**) analysis for P-ERK/T-ERK. β-actin was used as a loading control. The results are presented as mean ± SD from three repeated measurements using ImageJ version 1.54. *** *p* < 0.001 vs. unstimulated control.

To examine the effect of 1-acetyl-β-carboline on these pathways, we assessed ERK1/2 phosphorylation via Western blot. As shown in Figure 6, 1-acetyl-β-carboline reduced ERK phosphorylation by 20.79% at 12.5 μM and 25.63% at 25 μM. These results suggest that 1-acetyl-β-carboline promotes melanin synthesis by upregulating MITF and melanogenic enzymes, likely through modulation of the MAPK/ERK pathway.

### 2.7. 1-Acetyl-β-Carboline Is Safe for Human Skin

Human skin needs protection from environmental factors and chemicals found in pharmaceuticals and cosmetics. To safeguard the general population, especially vulnerable groups like children, it is crucial to assess the potential of cosmetic ingredients and final formulations to cause acute skin irritation. Consequently, a skin irritation test was performed to assess the effects of topical application of 1-acetyl-β-carboline. The primary irritant effects of two concentrations (25 μM and 50 μM) of 1-acetyl-β-carboline, dissolved in squalene, on human skin were evaluated. Testing was conducted according to the PCPC guidelines and Dermapro Inc. standard operating procedures. The patch test involved 32 women who met the inclusion and exclusion criteria, with a mean age of 43.03 ± 5.17 years. For the test, 20 μL of 1-acetyl-β-carboline was applied to the clean areas of the subjects’ backs for 24 h. Skin evaluations were performed 20 min and 24 h after removing the patches. The response to primary skin irritation was evaluated following the PCPC guidelines (Table 1), and the results showed that 1-acetyl-β-carboline exhibited hypoallergenic properties with respect to primary skin irritation (Table 2).

## 3. Materials and Methods

### 3.1. Media, Chemicals, and Antibodies

The bacterial media used in this study included actinomycete isolation agar (AIA; sodium caseinate 0.2%, asparagine 0.01%, sodium propionate 0.4%, dipotassium phosphate 0.05%, magnesium sulfate 0.01%, ferrous sulfate 0.0001%, agar 1.5%) and Bennett’s agar (yeast extract 0.1%, N-Z amine type A 0.2%, glucose 1%, beef extract 0.1%, agar 1.5%), both of which were purchased from KisanBio Co., Ltd. (Seoul, Korea). Additional media, including ISP2 (yeast-malt extract agar), ISP4 (inorganic salt starch casein agar), ISP5 (glycerol asparagine agar), and tryptic soy broth (TSB), were obtained from BD Biosciences (Franklin Lakes, NJ, USA). Dulbecco’s modified Eagle medium (DMEM) and penicillin/streptomycin (P/S) were supplied by Thermo Fisher Scientific (Waltham, MA, USA), while fetal bovine serum (FBS) was sourced from Merck Millipore (Burlington, MA, USA). Furthermore, 10× trypsin-EDTA (0.5%) was also obtained from Thermo Fisher Scientific (Waltham, MA, USA). α-melanocyte-stimulating hormone (α-MSH), protease/phosphatase inhibitor cocktail, sodium hydroxide (NaOH), L-3,4-dihydroxyphenylalanine (L-DOPA), cycloheximide, nalidixic acid, and arbutin were purchased from Sigma-Aldrich (St. Louis, MO, USA). Additionally, 3-(4,5-dimethylthiazol-2-yl)-2,5-diphenyltetrazolium bromide (MTT), dimethyl sulfoxide (DMSO), phosphate-buffered saline (PBS), and radioimmunoprecipitation assay (RIPA) buffer were sourced from Biosesang (Seongnam, Gyeonggi-do, Korea). The bicinchoninic acid (BCA) protein quantification kit was obtained from Thermo Fisher Scientific (Waltham, MA, USA). For Western blot experiments, 2× Laemmli sample buffer and Tween 20 were acquired from Bio-Rad (Hercules, CA, USA). Skim milk was purchased from BD Biosciences (Franklin Lakes, NJ, USA), and bovine serum albumin (BSA) was sourced from Bovostar (Bovogen, Melbourne, Australia). Additionally, 2-mercaptoethanol was purchased from Biobasic (Markham, ON, Canada). Sodium dodecyl sulfate (SDS), tris-buffered saline (TBS), and enhanced chemiluminescence (ECL) kits were supplied by Biosesang (Seongnam, Gyeonggi-do, Korea). For Western blotting experiments, primary antibodies for tyrosinase (#SC-20035), TRP-1 (#SC-166857), TRP-2 (#SC-74439), and MITF (#SC-25386) were purchased from Santa Cruz Biotechnology (Dallas, TX, USA), while β-actin (#7076S), P-ERK (Thr202/Tyr204, #9101S), ERK (#9101S), and secondary antibodies (anti-rabbit and anti-mouse, #7074S and #7076S) were acquired from Cell Signaling Technology (Danvers, MA, USA).

### 3.2. Isolation of Bacterial Strain and Culture Conditions

A soil sample was collected from Hwasun Gotjawal on Jeju Island (33°15′52.6″ N, 126°19′56.4″ E), Republic of Korea. A gram of soil was suspended in a saline solution, and serially diluted suspensions were seeded on AIA, Bennett’s agar, ISP2, and ISP5. To 1 L of all of the isolation media mentioned above, 100 μL of cycloheximide (50 mg/mL) dissolved in ethanol to inhibit the growth of fungi and 500 μL of nalidixic acid (10 mg/mL) dissolved in chloroform to inhibit the growth of common rapidly growing bacteria, such as *Bacillus* sp., were added, mandatorily to support selective isolation of actinomycetes. Agar plates were incubated at 30 °C for 7 days under aerobic conditions and *Lentzea* sp. strain. JNUCC 0626 was isolated from ISP5 medium. The growth of JNUCC 0626 was evaluated using AIA, Bennett’s agar, ISP2, ISP4, and ISP5. The strain was routinely subcultured in TSB broth at 30 °C and preserved in a 20% (*v*/*v*) glycerol suspension at −70 °C.

### 3.3. 16S rRNA Gene Sequence and Phylogenetic Analysis

Genomic DNA (gDNA) was extracted and purified from a single colony cultured on an ISP2 plate. The gDNA served as a template for amplifying the 16S rRNA gene using the universal bacterial primers 27F (5′-AGAGTTTGATCMTGGCTCAG-3′) and 1492R (5′-TACGGYTACCTTGTTACGACTT-3′), where M represents A or C. The purified PCR products were sequenced by Solgent Co. Ltd. (Daejeon, Korea). The full 16S rRNA gene sequences were assembled using SeqMan (DNASTAR, version 5.0) and Chromas (Technelysium, version 2.6.6) software. For phylogenetic tree construction, the sequences were aligned using the ClustalW tool. Phylogenetic trees were then generated using the maximum likelihood (ML) and neighbor-joining (NJ) algorithms, both executed in MEGA 11 software. Bootstrap values were analyzed with 300 or 1000 replications. The tree was rooted using *Umezawaea tangerina* MK27-91F2 as an outgroup. Genetic distances of the pairs were calculated using the Kimura two-parameter model with MEGA 11 software.

### 3.4. Isolation of 1-Acetyl-β-Carboline

#### 3.4.1. General Protocol

The solvents used for extraction, fractionation, and separation of individual compounds in the experiments were of analytical grade. High-performance liquid chromatography (HPLC) analyses were conducted using a sophisticated HPLC system. The system consisted of a Waters Alliance 2489 separation module, paired with a Waters 2695 UV/visible light detector, quaternary pump, column temperature control unit, and a Waters 717 plus autosampler (Milford, MA, USA). Data acquisition was managed using the Empower Pro data processing system (Waters Co., Milford, CT, USA). The elution conditions are outlined in Table 1. For structural identification, nuclear magnetic resonance (NMR) systems, including the JNM-LA 400 and JNM-ECX 400 (both FT-NMR systems from JEOL Co., Tokyo, Japan), were used. CD3OD, a specialized NMR solvent supplied by Cambridge Isotope Laboratories, Inc. (Tewksbury, MA, USA), was used as the NMR measurement solvent.

#### 3.4.2. Fermentation, Extraction, and Isolation

*Lentzea* sp. JNUCC 0626 strain was cultured in liquid TSB medium at 28 °C and 200 rpm for 7 days. A 5 mL aliquot of the seed culture was then inoculated into 12 flasks, each containing 700 mL of TSB medium supplemented with 0.5% tryptophan. The cultures were kept at 28 °C with shaking at 200 rpm for 7 days. Following a 10 L fermentation, the fermentation broth was centrifuged at 4000 rpm for 20 min to separate the mycelia from the supernatant. The supernatant was subsequently filtered and sequentially fractionated using hexane and ethyl acetate. This process yielded 19 mg of the hexane fraction and 380 mg of the ethyl acetate fraction. When methanol was added to the hexane fraction, insoluble precipitates were formed. These precipitates were separated by centrifugation at 13,000 rpm for 3 min. The resulting precipitate was then dissolved in chloroform, yielding a clear solution, which was subjected to HPLC analysis to assess purity. The compound was further identified by NMR.

### 3.5. Cell Culture and Cell Viability

Mouse B16F10 melanoma cells were obtained from the American Type Culture Collection (ATCC, Manassas, VA, USA) and cultured at 37 °C in a 5% CO_2_ atmosphere using DMEM supplemented with 1% penicillin/streptomycin and 10% FBS. The culture medium was replaced every 3 days, and subculturing was carried out when cell confluence exceeded 80%. For the melanogenic efficacy assay, cell viability was assessed by seeding B16F10 cells in 24-well plates at a density of 1 × 10^4^ cells per well, followed by preincubation at 37 °C with 5% CO_2_ for 24 h. Subsequently, the cells were treated with hexane or ethyl acetate fractions of *Lentzea* sp. JNUCC 0626, or with 1-acetyl-β-carboline, and incubated under the same conditions for 72 h. Subsequently, 0.2 mg/mL MTT reagent was added, and the cells were incubated for an additional 4 h. After removing the MTT-containing medium, the resulting purple formazan crystals were dissolved in 800 μL of DMSO per well. The dissolved formazan solution was then transferred in 100 μL aliquots to a 96-well plate, and absorbance was measured at 570 nm using a microplate spectrophotometer (Epoch, BioTek, CA, USA).

### 3.6. Measurement of Melanin Contents

The melanin content of the cultured cells can be quantified using spectrophotometry and expressed as the melanin content per cell or per culture area. B16F10 cells were seeded at a density of 8 × 10^4^ cells per 60 mm dish and incubated at 37 °C in a 5% CO_2_ atmosphere for 24 h. After incubation, cells were treated with concentrations determined to maintain a cell survival rate of >90%, as indicated by the MTT assay. The cells were incubated for an additional 72 h. The control group received no treatment, while the positive control group was treated with 100 nM α-MSH. After the 72 h incubation, the medium was removed, and the cells were washed twice with 1× PBS buffer. The cells were then lysed in RIPA buffer containing 1% protease inhibitor cocktail for 30 min at 4 °C, using 150 μL of lysis buffer per plate. The resulting lysate was transferred to 1.5 mL tubes and centrifuged at 15,000 rpm for 30 min at 4 °C to obtain a pellet. The supernatant was discarded, and the pellet was resuspended in 250 μL of 1 N NaOH containing 10% DMSO, then incubated for 20 min at 80 °C. The resulting solution was transferred to a 96-well plate (50 μL per well), and absorbance was measured at 405 nm using a microplate spectrophotometer (Epoch, BioTek, CA, USA).

### 3.7. Measurement of Intracellular Tyrosinase Activity

To evaluate the stimulatory effects of the hexane and ethyl acetate fractions of *Lentzea* sp. JNUCC 0626 extracts and 1-acetyl-β-carboline on tyrosinase activity, which plays a key role in melanogenesis by oxidizing tyrosine to dopaquinone, intracellular tyrosinase activity was measured. B16F10 cells were seeded at a density of 8 × 10^4^ cells per 60 mm dish and incubated at 37 °C in a 5% CO_2_ atmosphere for 24 h. After determining concentrations that maintained a cell survival rate > 90%, as verified by the MTT assay, cells were treated and incubated for an additional 72 h. The control group received no treatment, while the positive control group was treated with 100 nM α-MSH. After a 72 h incubation, the medium was removed, and the cells were washed twice with 1× PBS buffer. Cells were then lysed with 150 μL of RIPA buffer containing a 1% protease inhibitor cocktail per plate for 30 min at 4 °C. The lysates were collected using a cell scraper, transferred to 1.5 mL tubes, and centrifuged at 15,000 rpm for 30 min at 4 °C to obtain the supernatant. To quantify protein content, 10 μL of the supernatant was mixed with 200 μL of BCA reagent (prepared by combining reagents A and B in a 50:1 ratio) in a 96-well plate and incubated at 37 °C for 30 min according to the BCA protein assay kit protocol. Absorbance was measured at 562 nm using a microplate spectrophotometer (Epoch, BioTek, CA, USA), and a standard curve was generated using BSA. Equal amounts of protein from each sample were diluted and 20 μL of the protein solution was mixed with 80 μL of 2 mg/mL L-DOPA in a 96-well plate. After incubation at 37 °C for 1 h, the absorbance was measured at 490 nm using the same microplate reader to assess tyrosinase activity.

### 3.8. Western Blot

The Western blot experiments were conducted with modifications to the previous methodology to assess the activation of proteins involved in melanogenesis by 1-acetyl-β-carboline [29,41]. B16F10 cells were seeded at a density of 8 × 10^4^ cells per 60 mm dish and incubated at 37 °C in a 5% CO_2_ atmosphere for 24 h. Subsequently, the cells were treated with varying concentrations of 1-acetyl-β-carboline, ensuring that cell viability remained above 90%. Treatment durations were adjusted according to the expression time of the target protein. After incubation, the medium was removed, and the cells were washed twice with 1× PBS buffer. The cells were then lysed in 150 μL of lysis buffer per plate, consisting of RIPA buffer supplemented with a 1% protease inhibitor cocktail, and incubated at 4 °C for 20 min. The lysates were collected in 1.5 mL tubes and centrifuged at 15,000 rpm and 4 °C for 30 min to obtain the supernatant. Protein concentration was determined using the BCA protein assay as described earlier, with quantification based on a standard curve prepared with BSA. Equal amounts of protein from each sample were then mixed with 2× Laemmli sample buffer (in a 1:1 ratio) and heated at 100 °C for 3 min to prepare them for Western blotting. Once cooled, 16 μL of each sample was loaded onto an SDS-polyacrylamide gel for electrophoresis, allowing the proteins to be separated by size. The separated proteins were transferred onto a PVDF membrane using the Trans-Blot Turbo System. The membrane was blocked with 5% skim milk in 1× TBS-T for 1.5 h and then washed six times with 1× TBS-T at 10 min intervals. It was subsequently incubated overnight at 4 °C with primary antibodies diluted 1:2000 in 20 mL of 1× TBS-T. After washing, the membrane was incubated with secondary antibodies diluted 1:2000 in 1× TBS-T for 2 h at room temperature. After additional washes, the membrane was treated with an ECL kit to visualize the protein bands, which were detected using the ChemiDoc system (Bilvers-Lourmette, France).

### 3.9. Human Skin Patch Test

The ethical and scientific validity of this study was thoroughly reviewed by Dermapro’s Institutional Review Board (IRB No. 1-220777-A-N-01-B-DICN24054) according to the principles established in the Declaration of Helsinki. The study adhered strictly to ethical guidelines, including obtaining voluntary informed consent from all participants. Thirty-three female subjects, who met the inclusion and exclusion criteria, were enrolled in the study, with a mean age of 43.03 ± 5.17 years, ranging from 29–52 years. Before the study, the subjects were fully informed about its purpose, methods, and possible adverse events. Those who agreed to participate signed an informed consent form. The test site, located on the backs of the subjects, was cleaned with 70% ethanol before applying 20 μL of the test substance for a duration of 24 h. The first evaluation of the skin reaction was carried out 20 min after the removal of the applied substance, and the second evaluation took place 24 h later. Adverse reactions were assessed by a dermatologist at all evaluation points following the PCPC guidelines, utilizing both questionnaires and direct observation of the subjects. In cases where adverse reactions occurred, appropriate medical interventions or decisions were made at the discretion of the dermatologist, ensuring the safety and well-being of the subjects throughout the study. The skin reaction for each test substance was calculated using a predefined formula, and the average reactivity of each substance was determined on the basis of these calculations. However, according to the Personal Care Products Council (PCPC) guidelines, if a skin reaction is graded +5, it is considered more likely to be an allergic reaction than an irritant one; therefore, the maximum possible grade was limited to +4 in this study.

$$\mathrm{Response}=\frac{\sum \left(Grade\times No.\;of\;Responders\right)}{4\;(Maximum\;Grade)\times n\;(Total\;Subjects)}\times 100\times 1/2$$

### 3.10. Statistical Analyses

Statistical analysis was performed using IBM SPSS (v.20, SPSS Inc., Armonk, NY, USA) with Student’s *t* test or one-way analysis of variance (ANOVA). All experimental results are expressed as the mean ± standard deviation (SD), based on at least three independent experiments or a single experiment conducted in triplicate. Statistical significance is indicated as *** *p* < 0.001 compared to the control.

## 4. Conclusions

We successfully isolated the rare actinomycete strain *Lentzea* sp. JNUCC 0626 from Hwasun Gotjawal, a biologically diverse volcanic lava field on Jeju Island. Phylogenetic analysis based on a partial 16S rRNA gene sequence (1480 bp), using MEGA 11, identified *Lentzea cavernae* SYSU K10001 as its closest relative. The hexane fraction, cultured in TSB with 1% tryptophan for more than seven days and extracted with an organic solvent, significantly enhanced melanin production, leading to the first report of isolation of 1-acetyl-β-carboline from *Lentzea* sp. Its melanogenic efficacy was further confirmed in B16F10 cells, where 1-acetyl-β-carboline activated MITF and upregulated key melanogenic enzymes, including tyrosinase, TRP-1, and TRP-2. Furthermore, a skin patch test demonstrated its safety for topical application, suggesting its potential use in cosmetics and ointments. 

This study highlights the significance of isolating rare actinomycetes for novel dermatological applications while also demonstrating the potential of 1-acetyl-β-carboline to be developed as a therapeutic or cosmetic agent for treating hypopigmentation-related conditions, with promising implications for human health.

## Figures and Tables

**Table 1:** Grading system for the primary skin irritation test.

Grade	Description of Clinical Observations
+1	Slight erythema
+2	Moderate erythema, possibly with barely perceptible edema at the margin, papules may be present.
+3	Moderate erythema, with generalized edema
+4	Severe erythema with severe edema, with or without vesicles
+5	The severe reaction spread beyond the area of the patch

**Table 2:** Results of the human skin primary irritation test (*n* = 32).

No	Test Sample	No. of Responder	1st Assessment				2nd Assessment				Reaction Grade (R) *
			+1	+2	+3	+4	+1	+2	+3	+4	
1	1-acetyl-β-carboline (25 μM)	0	0	0	0	0	0	0	0	0	0
2	1-acetyl-β-carboline (50 μM)	0	0	0	0	0	1	0	0	0	0

* None to slight: 0.00 ≤ R < 0.87.

## Data Availability

The authors confirm that all the data needed to support the study are presented within the article.
